# Probing the Structural and Electronic Properties of Dirhenium Halide Clusters: A Density Functional Theory Study

**DOI:** 10.1038/s41598-018-25027-1

**Published:** 2018-04-30

**Authors:** Li Huan Zhang, Xin Xin Xia, Wei Guo Sun, Cheng Lu, Xiao Yu Kuang, Bo Le Chen, George Maroulis

**Affiliations:** 1 Institute of Atomic and Molecular Physics, Sichuan University, Chengdu, 610065 China; 2 Department of Physics, Nanyang Normal University, Nanyang, 473061 China; 3 Department of Physics and High Pressure Science and Engineering Center, University of Nevada, Las Vegas, Nevada 89154 United States; 4 Department of Chemistry, University of Patras, GR-26500 Patras, Greece

## Abstract

Dirhenium halide dianions received considerable attention in past decades due to the unusual metal–metal quadruple bond. The systematic structural evolution of dirhenium halide clusters has not been sufficiently studied and hence is not well-understood. In this work, we report an in-depth investigation on the structures and electronic properties of doubly charged dirhenium halide clusters Re_2_X_8_^2−^ (X = F, Cl, Br, I). Our computational efforts rely on the well-tested unbiased CALYPSO (Crystal structure AnaLYsis by Particle Swarm Optimization) method combined with density functional theory calculations. We find that all ground-state Re_2_X_8_^2−^ clusters have cube-like structures of *D*_4*h*_ symmetry with two Re atoms encapsulated in halogen framework. The reasonable agreement between the simulated and experimental photoelectron spectrum of the Re_2_Cl_8_^2−^ cluster supports strongly the reliability of our computational strategy. The chemical bonding analysis reveals that the δ bond is the pivotal factor for the ground-state Re_2_X_8_^2−^ (X = F, Cl, Br, I) clusters to maintain *D*_4*h*_ symmetric cube-like structures, and the enhanced stability of Re_2_Cl_8_^2−^ is mainly attributed to the chemical bonding of 5d orbital of Re atoms and 3p orbital of Cl atoms.

## Introduction

The discovery^1^ of the metal–metal quadruple bond in dirhenium halide Re_2_Cl_8_^2−^, which is recognized as an important milestone in the development of modern inorganic chemistry, has stimulated intensive research work in the field of transition metal chemistry^2,3^. Understanding the properties of the metal–metal multiple bond is significant to further elucidate the chemistry of transition metal complexes. As a prototypical metal–metal multiple bonded ion, Re_2_Cl_8_^2−^ has been studied extensively in the past decades^2,4–22^. The experimental and theoretical investigations mainly focused on the preparation^4–6^, the molecular structure^7,8^, the metal–metal multiple bond^2,9–14^ and the electronic properties^4,10,15–22^ of Re_2_Cl_8_^2−^ in the octachlorodirhenate salts. Previous studies^7–13^ have determined the geometry structure of Re_2_Cl_8_^2−^ to be of *D*_4*h*_ symmetry, and confirmed the formation of Re–Re quadruple bond is due to the σ^2^π^4^δ^2^ subset of the bonding orbitals. Due to the difficulty of preparing the Re_2_Cl_8_^2−^ dianion in the gas phase, Wang *et al*.^21^ first measured the photoelectron spectrum (PES) of gaseous Re_2_Cl_8_^2−^ several decades after the discovery of the metal−metal multiple bond, leading to a thorough understanding of the Re–Re quadruple bond and its electronic structure.

Compared to the extent of the investigations on Re_2_Cl_8_^2−^, similar treatments of the heavier halogen derivative Re_2_Br_8_^2−^, which contains the Re–Re quadruple bond as well, are limited^4,17,23,24^. To our knowledge, sparse studies have been reported on the lighter halogen derivative Re_2_F_8_^2−^. Peters *et al*.^25^ initially synthesized (*n*-Bu_4_N)_2_[Re_2_F_8_]·4H_2_O, which contains Re_2_F_8_^2−^, via Re_2_Cl_8_^2−^ and (*n-*Bu_4_N)F·3H_2_O reacting in CH_2_Cl_2_ solution. Conradson *et al*.^26^ later confirmed the presence of the Re–Re quadruple bond in Re_2_F_8_^2−^ using X-ray absorption fine structure and resonance Raman spectroscopy. The structure of Re_2_F_8_^2−^ was determined to be eclipsed *D*_4*h*_ geometry by Henkel *et al*.^27^. Recently, Balasekaran *et al*.^28^ prepared the (NH_4_)_2_[Re_2_F_8_]·2H_2_O salt in nearly 90% yield by the reaction of (*n*-Bu_4_N)_2_[Re_2_Cl_8_] with molten NH_4_HF_2_. They also investigated the molecular and electronic structure along with the electronic absorption spectrum of Re_2_F_8_^2−^ relying on multiconfigurational quantum chemical calculations. Compared to Re_2_F_8_^2−^, studies on Re_2_I_8_^2−^ are sparse as well. Glicksman *et al*.^29^ firstly reported a synthesis route of Re_2_I_8_^2−^ via the reaction of the dirhenium complex Re_2_(O_2_CC_6_H_5_)_4_Cl_2_ and HI with Bu_4_N^+^ in ethanol or methanol to produce the (Bu_4_N)_2_Re_2_I_8_ salt. Preetz *et al*.^30^ prepared (Bu_4_N)_2_Re_2_I_8_ successfully for the first time in a simple procedure by the reaction of (Bu_4_N)_2_Re_2_X_8_ with HI in dichloromethane. Preetz *et al*.^31^ also reported measurements of the resonance Raman spectrum of (Bu_4_N)_2_Re_2_I_8_, while Cotton *et al*.^32^ studied its crystal structure until a few years later. Extensive studies on Re_2_X_8_^2−^ have been reported, but almost all previous studies focused on Re_2_X_8_^2−^ dianions in crystals. There has been no systematic work on Re_2_X_8_^2−^ (X = F, Cl, Br and I) clusters until now, so the following questions attract our interests: (i) It is not clear whether Re_2_X_8_^2−^ clusters are characterized by the same molecular geometry as Re_2_X_8_^2−^ dianions in crystals. (ii) Does the chemical bonding in Re_2_X_8_^2−^ clusters differ from that of dianions in crystals? (iii) What is the relative stability of Re_2_X_8_^2−^ clusters? Consequently, we turn our attention to the systematic study of lowest-energy geometries and electronic structures of Re_2_X_8_^2−^ (X = F, Cl, Br, I) clusters.

As a first step of our study on the structural evolution of Re_2_X_8_^2−^ clusters, we perform a search for the the low-energy structures of Re_2_X_8_^2−^ (X = F, Cl, Br, I) by means of the CALYPSO (Crystal structure AnaLYsis by Particle Swarm Optimization) code combined with DFT (density functional theory) calculations. We get the ground-state structures of Re_2_X_8_^2−^ clusters from the above calculations and subsequently investigate their relative stabilities and chemical bonding. This paper is organized as follows. The obtained results and a pertinent discussion are in the following. Then we summarize our conclusions. Last, a detailed presentation of the computational method is described.

## Results and Discussion

### Geometric Structure

**Figure 1:**
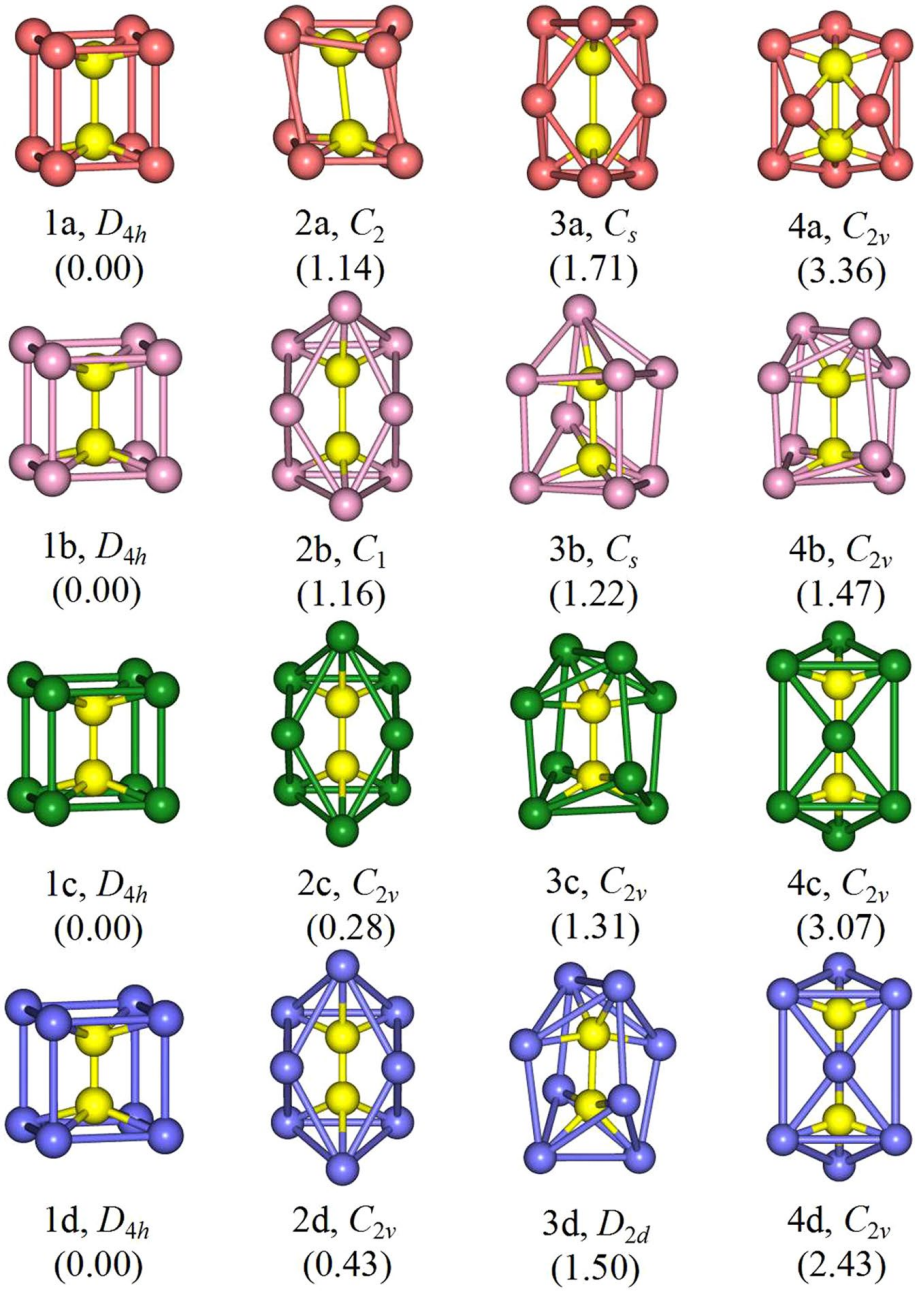
The geometrical structures of low-lying Re_2_X_8_^2−^ (X = F, Cl, Br, I) clusters, along with the point group symmetry and relative energy (eV). Rhenium atoms are in yellow. Fluorine, chlorine, bromine and iodine atoms are in orange, pink, green and purple, respectively.

The lowest-energy structures of the Re_2_X_8_^2−^ (X = F, Cl, Br, I) clusters, together with other three typical low-lying isomers are shown in Fig. 1. The optimized vibrational frequencies and atomic coordinates of ground-state Re_2_X_8_^2−^ (X = F, Cl, Br, I) clusters are all collected in Table S1 and Table S2 of the supplementary information. According to their energies from low to high, all isomers are denoted by na, nb, nc and nd (n = 1, 2, 3,4), in which a, b, c and d stand for Re_2_F_8_^2−^, Re_2_Cl_8_^2−^, Re_2_Br_8_^2−^ and Re_2_I_8_^2−^, respectively. The electronic states, point group symmetries, average binding energies, HOMO−LUMO (the highest occupied molecular orbitals and the lowest unoccupied molecular orbitals) energy gaps, together with charge on Re atoms and halogen atoms of the ground-state Re_2_X_8_^2−^ clusters are summarized in Table 1. In addition, the electronic states, point group symmetries, relative energies, HOMO−LUMO energy gaps of all isomers are given in Table S3.

**Table 1:** Electronic states, point group symmetries, average binding energies *E*_b_ (eV), HOMO−LUMO energy gaps *E*_gap_ (eV) along with charge *Q* (e) on Re atoms and halogen atoms of Re_2_X_8_^2−^ (X = F, Cl, Br, I) clusters.

Cluster	State	Sym.	*E*_b_	*E*_gap_	*Q* (Re)	*Q* (X)
Re_2_F_8_^2−^	^1^A_1g_	*D*_4*h*_	3.91	1.94	1.29	−0.57
Re_2_Cl_8_^2−^	^1^A_1g_	*D*_4*h*_	2.49	1.71	−0.03	−0.24
Re_2_Br_8_^2−^	^1^A_1g_	*D*_4*h*_	2.07	1.66	−0.32	−0.17
Re_2_I_8_^2−^	^1^A_1g_	*D*_4*h*_	1.72	1.43	−0.60	−0.10

It can be seen in Fig. 1 that the ground state 1a, 1b, 1c and 1d of Re_2_X_8_^2−^ (X = F, Cl, Br, I) all possess cube-like geometric structures with *D*_4*h*_ symmetry. The geometric structures of ground-state Re_2_X_8_^2−^ (X = F, Cl, Br, I) clusters favor low-spin ^1^A_1g_ state and have the same symmetry with those in crystals^2,7,11,23,27,32^. With the increase of the halogen atom size, the Re–Re bond length in Re_2_X_8_^2−^ varies from Re_2_F_8_^2−^ to Re_2_I_8_^2−^ as 2.20 Å, 2.21 Å, 2.22 Å, 2.24 Å, showing a little difference to those of Re_2_X_8_^2−^ dianions in crystal^2^. The 2a isomer has a distorted cube-like structure with *C*_2_ symmetry and the energy is 1.14 eV higher than its ground-state structure 1a. The metastable isomer 3a of Re_2_F_8_^2−^ has *C*_*s*_ symmetry, at 1.71 eV higher than its global minimum 1a. Interestingly enough, the structure of 3a is a small square-bottom boat, and the two rhenium atoms are located in its interior. The point group symmetry of the 4a isomer is *C*_2*v*_, and its energy is 3.36 eV higher than its lowest-energy structure 1a. It is obvious that the two rhenium atoms are exposed outside in the geometry structure of 4a. It can be clearly seen from Fig. 1 that 2b has similar structures with 3a. The isomer of 2b has *C*_1_ symmetry with its energy 1.16 eV higher than the ground-state structure 1b. The isomers of 3b and 4b display cage-like structure with the two Re atoms encapsulated in a chlorine framework. They possess *C*_*s*_ and *C*_2*v*_ symmetry with the energy 1.22 eV and 1.47 eV, respectively, higher than their lowest-energy structures 1b. The isomer 2c has *C*_2*v*_ symmetry, with total energy 0.28 eV higher than the ground-state 1c, whose structure is similar to that of 2b. The 3c structure of *C*_2*v*_ symmetry looks similar to the structure of 4b, with total energy higher than 1c by 1.31 eV. The *C*_2*v*_ symmetry 4c isomer is 3.07 eV less stable than the ground-state 1c. The 2d isomer of *C*_2*v*_ symmetry has similar structure to the isomers 2b and 2c with the total energy 0.43 eV higher than 1d structure. The structure of 3d, which possesses *D*_2*d*_ symmetry with total energy higher than the 1d one by 1.50 eV, is similar to that of 4b and 3c isomers. The *C*_2*v*_ symmetry 4d isomer, at 2.43 eV higher in energy than the ground-state 1d, has similar structure to 4c. It is worth noting that, except for the 4a structure of Re_2_F_8_^2−^, the two rhenium atoms are invariably inclined to stay inside for all ground and excited state structures of Re_2_X_8_^2−^ (X = F, Cl, Br, I). In order to confirm the validity of our calculations, we have also optimized the Re_2_X_8_^2−^ (X = F, Cl, Br, I) clusters at the BP86 level of theory. The corresponding lowest-energy geometries of Re_2_X_8_^2−^ clusters are presented in Figure S1 of the supplementary information. From Figure S1 we can see all structures optimized at BP86 functional also have cube-like structures with *D*_4*h*_ symmetry and the same electronic state of ^1^A_1g_. The respective ground-state structures are in agreement with those obtained by our B3LYP optimization, further supporting our claim for the reliability of our theoretical approach.

### Photoelectron Spectra of Re_2_X_8_^2−^

**Figure 2:**
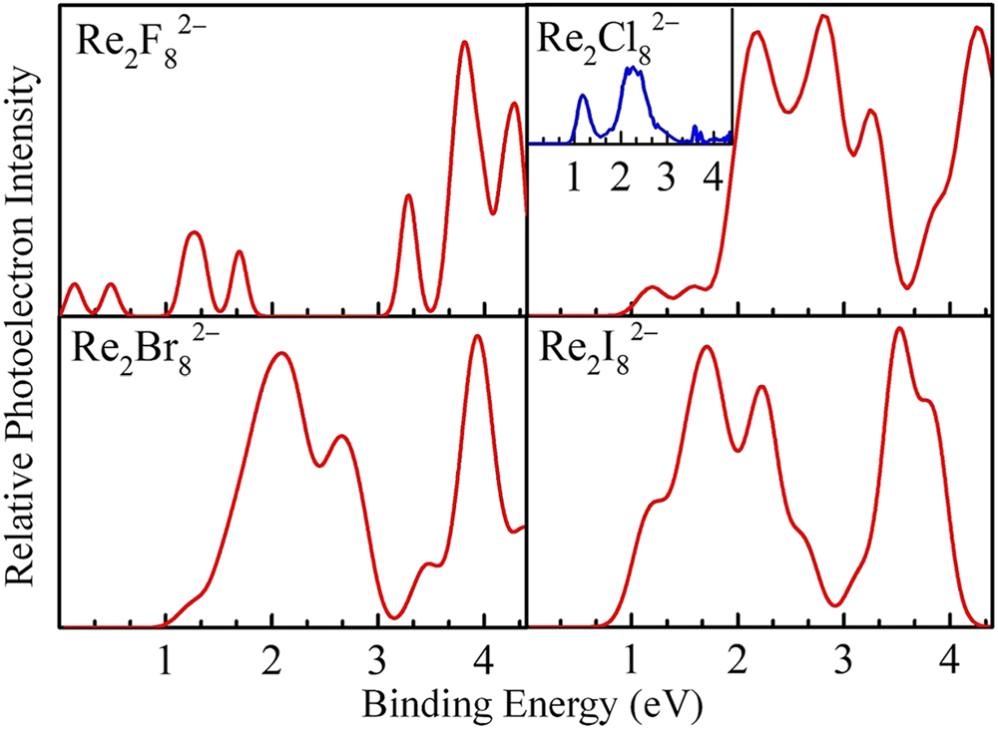
The simulated photoelectron spectra of the ground-state structures of Re_2_X_8_^2−^ (X = F, Cl, Br, I) clusters, along with the available experimental spectrum of Re_2_Cl_8_^2−^ in the inset.

Photoelectron spectrum (PES) is a powerful tool, which can provide important information about the electronic configuration of ground-state structures. In order to verify the validity of our obtained structures in this work, as shown in Fig. 2, we have simulated the photoelectron spectra of all global minimum geometries of Re_2_X_8_^2−^ (X = F, Cl, Br, I) and compared to the available experimental PES. Moreover, the values of adiabatic detachment energy (ADE), extracted from the threshold of the first peak, and the values of vertical detachment energy (VDE), obtained from the first peak maximum of the spectra, are summarized in Table S5 together with the available experimental data^21^.

As shown in Fig. 2, all spectra exhibit rich features, which correspond to transitions from the ground state of Re_2_X_8_^2−^ to the ground and excited states of singly charged Re_2_X_8_^−^. Overall, the binding energies, spacings and intensities are different in each spectrum, but the spacings and intensities of spectra features for Re_2_Br_8_^2−^ and Re_2_I_8_^2−^ are nearly similar to each other. The most prominent spectral feature of Re_2_F_8_^2−^ is the observation of a lower adiabatic detachment energy measured as 0.05 eV. This phenomenon is normal for multiply charged anions, largely due to the presence of intramolecular Coulomb repulsion^33,34^. It can be clearly seen that the first two peaks of the Re_2_F_8_^2−^ spectrum are very weak and appear in very lower binding energies. The following relatively intense peaks locate at 1.25 and 1.70 eV, respectively, and the most intense peak locates at 3.80 eV. For the simulated PES of Re_2_Cl_8_^2−^, the ADE, namely the second electron binding energy, is 0.98 eV, which is consistent with the experimental datum of 1.00 eV. The first peak is located at 1.19 eV, in reasonable agreement with the first peak location of the experimental spectrum of Re_2_Cl_8_^2−^ centered at 1.16 eV. The following three peaks are relatively intense, located at 2.21, 2.80, and 3.24 eV, respectively. In the simulated PES of Re_2_Br_8_^2−^, the first peak is quite intense, and emerges at 2.09 eV. In addition, there is a relatively sharp peak, which is the most intense one, located at 3.95 eV. The simulated spectrum of Re_2_I_8_^2−^ exhibits three obviously intense peaks and the first relatively weak peak emerges at 1.21 eV. The agreement of the ADE and VDE values of the simulated and experimental PES for Re_2_Cl_8_^2−^ supports the validity of our computational approach. We hope that the simulated PES of dirhenium halides can provide a helpful reference for the spectroscopic studies of Re_2_X_8_^2−^ (X = F, Br, I) dianions.

### Relative Stabilities

To explore the relative stabilities of the ground state structures of the dirhenium halide system, we have calculated the average binding energies (*E*_b_). For Re_2_X_8_^2−^ (X = F, Cl, Br, I) clusters, the *E*_b_ can be defined as follow:

1 $${E}_{{\rm{b}}}=[2E({\rm{Re}})+6E({\rm{X}})+2E({{\rm{X}}}^{-})-E({{\rm{Re}}}_{2}{{{\rm{X}}}_{8}}^{2-})]/10$$

**Figure 3:**
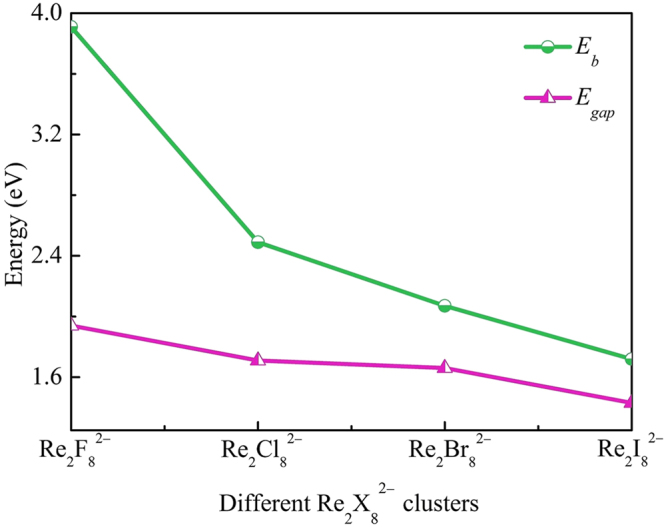
The average binding energies per atom *E*_b_ and HOMO−LUMO energy gaps of Re_2_X_8_^2−^ (X = F, Cl, Br, I).

Where *E*(Re_2_X_8_^2−^), *E*(Re), *E*(X), and *E*(X^−^) represent the energy of Re_2_X_8_^2−^ clusters, neutral Re atom, neutral X atom, and anionic X^−^, respectively. The average binding energies of Re_2_X_8_^2−^ (X = F, Cl, Br, I) clusters are displayed in Fig. 3. From Fig. 3 we can find that Re_2_F_8_^2−^ is relatively stable due to its large average binding energy value. Besides, the *E*_b_ values of Re_2_X_8_^2−^ decrease monotonically from Re_2_F_8_^2−^ to Re_2_I_8_^2−^, indicating that the relative stabilities of Re_2_X_8_^2−^ (X = F, Cl, Br, I) decrease with increasing size of the halogen atom.

Another important parameter related to the relative stabilities of the Re_2_X_8_^2−^ clusters is the HOMO−LUMO energy gap (*E*_gap_), which represents the ability of an electron to jump from the highest occupied molecular orbital (HOMO) to the lowest unoccupied molecular orbital (LUMO). A large value of *E*_gap_ implies the corresponding molecular structure is chemically inert. The evolution of the HOMO−LUMO energy gap for the ground-state Re_2_X_8_^2−^ clusters is shown in Fig. 3. It is clearly seen that the *E*_gap_ decrease monotonically, that is to say, the clusters are relatively less reactive as the halogen atom size increases, in agreement with the obtained results for the average binding energies. However, in contrast to the average binding energy rapid decrease from Re_2_F_8_^2−^ to Re_2_I_8_^2−^, the HOMO−LUMO energy gap decreases less steeply.

### Charge Transfer

Electronegativity^35–39^ is an important chemical property to describe the ability of an atom to attract electrons. A large electronegativity value is interpreted as a strong ability to attract electrons. In order to investigate the charge transfer between Re atoms and halogen atoms for ground-state Re_2_X_8_^2−^ clusters and compare to the electronegativity, we utilize natural population analysis (NPA) to obtain the charge on each atom. The results are summarized in Table 1. From Table 1 we can see that the negative charge on halogens gradually decreases from Re_2_F_8_^2−^ to Re_2_I_8_^2−^. This phenomenon agrees with the decreasing values of Pauling electronegativity, which are 3.98, 3.16, 2.96 and 2.66^40^ for F, Cl, Br and I, respectively. Meanwhile, the charge on Re changes from positive to negative and the negative charge on Re is increasing with the halogen atom size increasing. All this indicates that it becomes more competitive for Re to attract electrons as the electronegativity values of halogens decrease. Although the electronegativity value of Re (1.93 on the Pauling scale)^36^ is lower than that of all halogen atoms, the negative charge on Re atoms is more than that of Br and I for Re_2_Br_8_^2−^ and Re_2_I_8_^2−^, clearly not in accord with the electronegativity. This is perhaps related to the weak ability of Br and I halogen atoms to attract electrons caused by their large atomic radii and the strong intramolecular Coulomb repulsion observed in multiply charged anions^34^. In summary, the two excess negative charges shift from peripheral halogens to Re atoms in the case of heavier halogen atoms.

### Chemical Bonding Analysis

**Figure 4:**
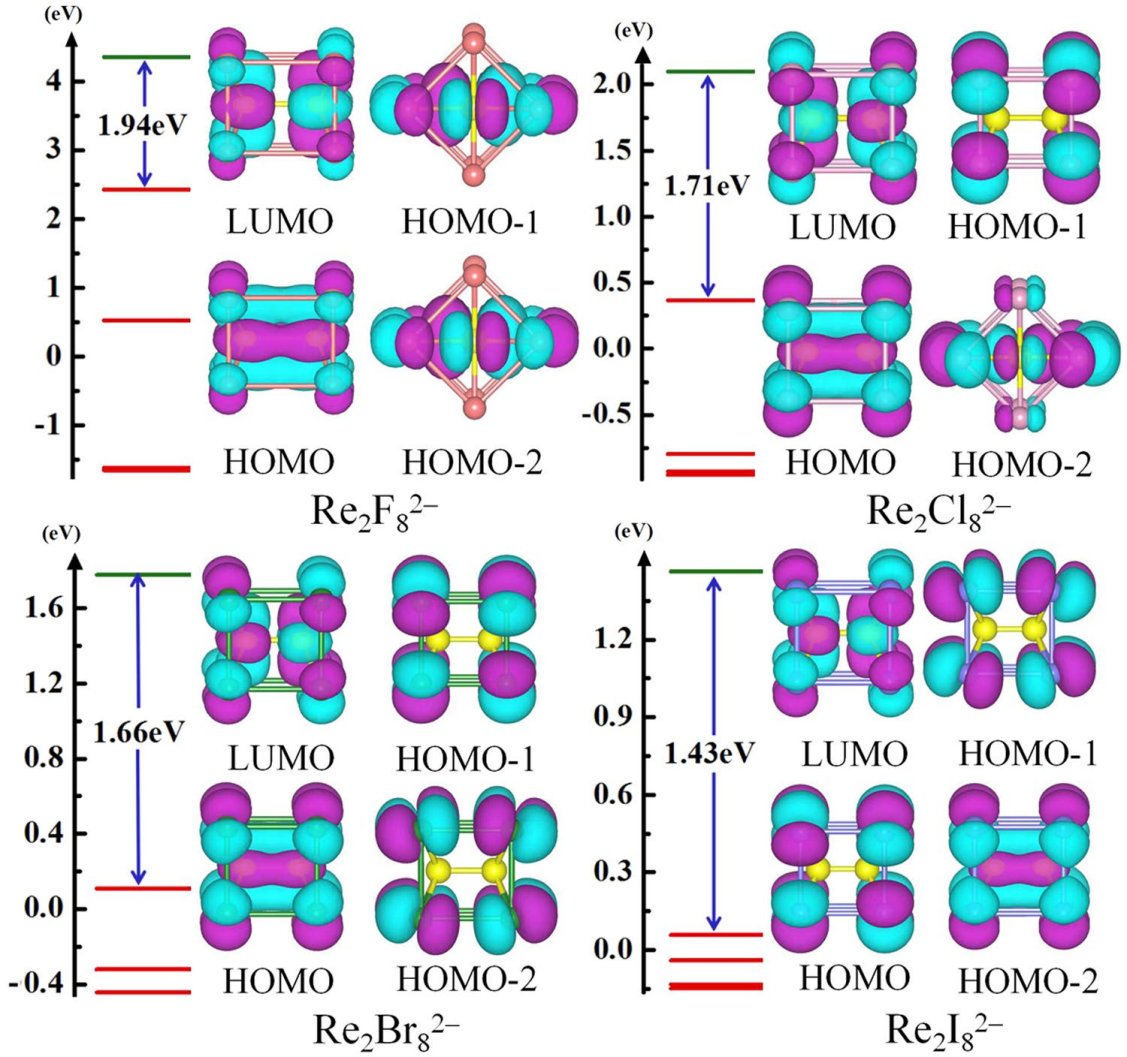
Molecular orbital maps and energy levels of Re_2_X_8_^2−^ (X = F, Cl, Br, I). The HOMO−LUMO energy gap is indicated (in blue).

In order to explore the nature of the chemical bonding in Re_2_X_8_^2−^, we have carried out a detailed analysis on the HOMO−LUMO molecular orbitals (MOs). Figure 4 shows the molecular orbital diagrams of Re_2_X_8_^2−^ clusters together with their orbital energy levels, which feature their HOMO−LUMO energy gaps. Herein we take the Re–Re bond as *z* axis and the Re–Cl bond as ± *x* and ± *y* axes to analyze the chemical bonding. As shown in Fig. 4, the HOMO of Re_2_F_8_^2−^ displays a large overlap between the 5d_*xy*_ orbitals of the two Re atoms, and the overlap forms a δ bond. The HOMO–1 and HOMO−2 are doubly degenerate, which feature π bonds formed by the overlap of the 5d_*yz*_ orbitals. From the HOMO of Re_2_Cl_8_^2−^, it can be seen that the 5d_*xy*_ orbitals of the Re atoms overlap and this leads to the formation of the δ bond. In Re_2_Cl_8_^2−^, the HOMO-2 and HOMO–3 are doubly degenerate, leading to π bonds formation by the overlap of the 5d_*yz*_ orbitals. In the HOMO of Re_2_Br_8_^2−^, the 5d_*xy*_ orbitals of Re atoms have an overlap, leading to the formation of the δ bond. As for Re_2_I_8_^2−^, there is no bonding between Re and Re in HOMO, while the HOMO-2 reveals an overlap between the 5d_*xy*_ orbitals of the Re atoms, forming a δ bond. Both the HOMO of Re_2_X_8_^2−^ (X=F, Cl, Br) and the HOMO−2 of Re_2_I_8_^2−^ are δ bonds. Compared to σ and π bonds^10,13,21^, the δ bond is weak and contributes relatively a little to the total strength of the Re–Re bond. Nevertheless, it plays a vital role in geometric structure. The δ bond strength achieves its maximum only when the conformation is eclipsed. Its strength reduces as the conformation becomes staggered^19^. Thus, we can deduce that the δ bond is the pivotal factor for the ground-state Re_2_X_8_^2−^ (X = F, Cl, Br, I) clusters to maintain *D*_4*h*_ symmetric cube-like structures.

**Figure 5:**
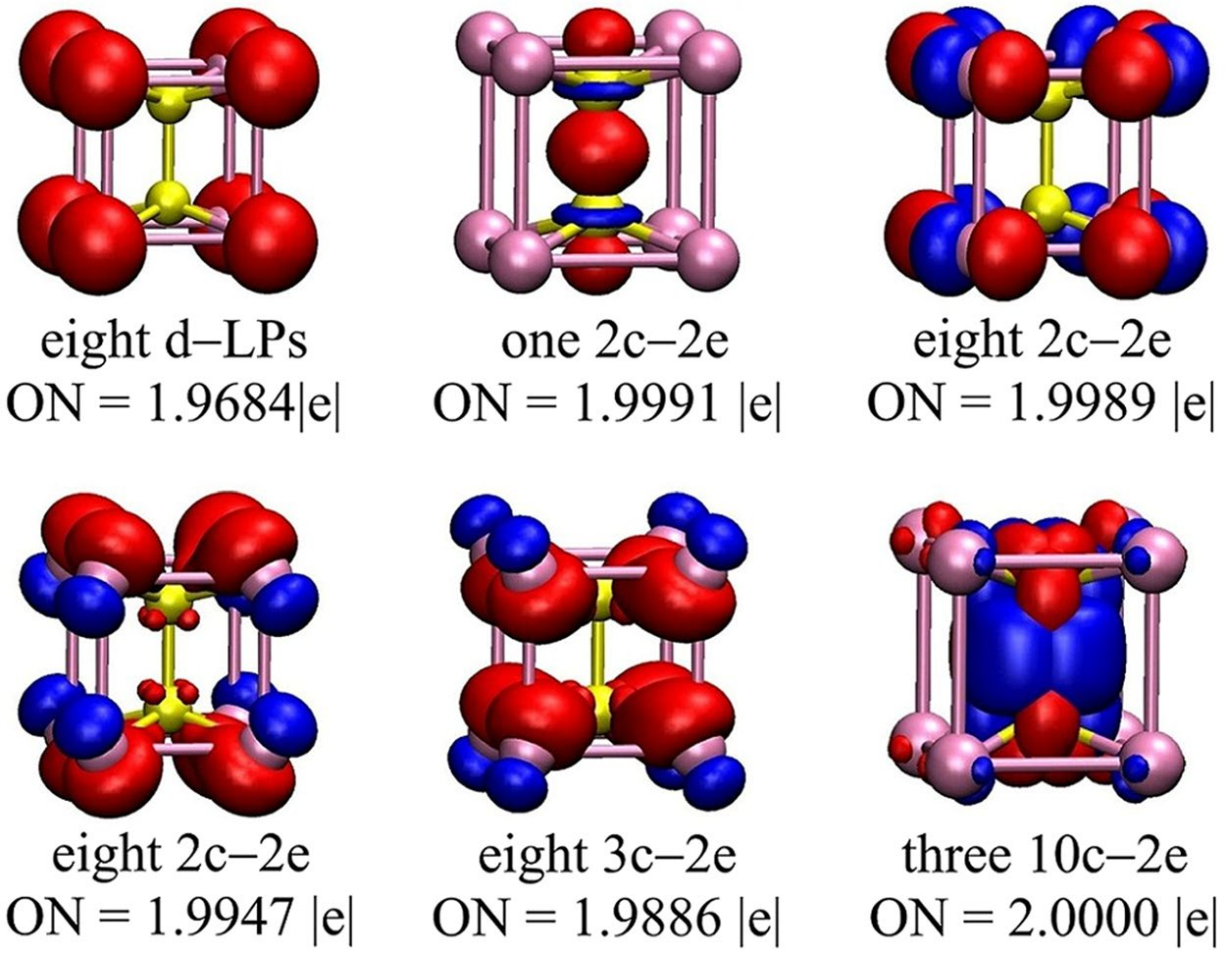
The results of AdNDP analysis for Re_2_Cl_8_^2−^. ON represents the occupation number.

To further understand the bonding mechanism in Re_2_X_8_^2−^, we select Re_2_Cl_8_^2−^ as a representative example in order to perform a chemical bonding analysis using the adaptive natural density partitioning (AdNDP) method, which is particularly suitable to decipher the nature of the chemical bond. The AdNDP results displayed in Fig. 5 reveals 25 localized bonds and 11 delocalized bonds with occupation numbers (ONs) ranging from 1.9684 to 2.0000 |e|, very close or nearly equal to the ideal values (2.000 |e|). There are eight 1c–2e lone pairs (3 s) with the ON of 1.9684 |e|. The seventeen 2c–2e bonds can be divided into three sets, which contain one Re–Re bond (ON = 1.9991 |e|), eight Cl–Cl bonds (ON = 1.9989 |e|), and eight Re–Cl bonds (ON = 1.9947 |e|), respectively. The Re–Re bond is formed by the overlap of 5d orbital between the two Re atoms. For the Cl–Cl bonds, the 3p orbital in the peripheral Cl atoms results in the formation of the 2c–2e bonds. The interaction between Re and Cl is mainly dominated by the 5d orbital of Re atom and the 3p orbital of the Cl atom. The eight 3c–2e bonds with ON = 1.9886 |e| are formed by the 5d orbitals of two Re atoms and the 3p orbital of one Cl atom. There are three 10c−2e bonds with the ONs of 2.0000 |e| between the two Re atoms and the Cl_8_ framework, which strengthen the interactions between the inside Re atoms and the peripheral Cl atoms. In addition, the high occupation number among the localized and delocalized bonds may point to its stability. Overall, the enhanced stability of Re_2_Cl_8_^2−^ is mainly caused by the chemical bonding of 5d orbital of Re atoms and 3p orbital of Cl atoms.

## Conclusions

We have performed a comprehensive research on the structural and electronic properties of dirhenium halide clusters via unbiased CALYPSO structure searches combined with DFT optimizations. The results clearly reveal that the ground states of Re_2_X_8_^2−^ (X = F, Cl, Br, I) all possess cube-like geometry with *D*_4*h*_ symmetry. This finding has been further tested by performing calculations at the BP86 level. The simulated PES of Re_2_Cl_8_^2−^ shows reasonable agreement with the experimental spectrum, which further confirms the validity of our computational approach. According to the average binding energy and HOMO−LUMO energy gap, Re_2_X_8_^2−^ clusters are progressively less stable as halogen atoms become heavier. The chemical bonding analysis indicates that the δ bond is the pivotal factor for the ground-state Re_2_X_8_^2−^ (X = F, Cl, Br, I) clusters to retain *D*_4*h*_ symmetric cube-like structures, and the enhanced stability of Re_2_Cl_8_^2−^ is mainly due to the chemical bonding of 5d orbital of Re atoms and 3p orbital of Cl atoms. We expect our findings provide stimulations and guidance to researchers for further experimental or theoretical investigations on binuclear transition metal halide clusters.

## Computational Method

The low-lying isomers of Re_2_X_8_^2−^ (X = F, Cl, Br, I) clusters are determined via the CALYPSO method which is based on the particle swarm optimization (PSO) algorithm. This theoretical method has been described in sufficient detail previously^41–43^. In brief, it can explore the global minima of the potential energy surfaces of predicted system only based on the chemical compositions at given external conditions. It has achieved great success in predicting the ground-state structures of various systems^44–49^. Herein, initial structures for Re_2_X_8_^2−^ clusters are achieved by the CALYPSO method. Each generation contains 50 structures and 60% of those are generated by PSO, while the other structures are generated randomly. We have followed 30 generations for each cluster to achieve convergence to the global minima on the potential energy surfaces. The candidates within 4 eV of the lowest-energy structure are further optimized using the B3LYP^50,51^ exchange-correlation functional. The 6-311 + G(d) basis set for F and the LANL2DZ^52,53^ basis with effective core potentials for Cl, Br, I and transition-metal Re are employed throughout all calculations. All these calculations are carried out using the Gaussian 09 software package^54^. The choice of the B3LYP functional and The LANL2DZ basis leans heavily on experience acquired from the previous investigations^11,13,55^. Different spin multiplicities are taken into consideration in the geometric optimization process (up to quintet for all clusters). The harmonic vibrational frequencies are calculated in order to ensure that the optimized structures are true minima without any imaginary frequencies. We also perform the natural bond orbital (NBO)^56^ and adaptive natural density partitioning (AdNDP)^57^ to get further insight into the chemical bonding mechanism.

In order to identify the reliability of our calculations, we have optimized the structures of dirhenium halide clusters with the non-hybrid BP86 functional and the same basis set as above. It is readily seen that the optimized results of Re_2_X_8_^2−^ clusters obtained by BP86 method are in accordance with our B3LYP results. In addition, compared with the Re–Re bond length of Re_2_X_8_^2−^ dianions in previous study^2^ (Table S4), we can see the results calculated by the B3LYP level agree better than those from the BP86 functional. Thus we conclude that the results obtained at B3LYP level are reliable. To further confirm the reliability of our computational method, we simulate the photoelectron spectra (PES) of all lowest-energy dirhenium halide clusters and compare to the available experimental PES^21^. The first VDE is obtained based on the energy difference between the singly charged anion and dianion at the ground-state geometry of the dianion. The excited-state energies of the singly charged anion are calculated via the time-dependent density functional theory (TD-DFT) method^58^. The reasonable agreement between the simulated and the experimental PES of Re_2_Cl_8_^2−^ strongly supports our choice of B3LYP functional.

## Electronic supplementary material

Supplementary information
