# Supplementary material for: Probing the Structural and Electronic Properties of Dirhenium Halide Clusters: A Density Functional Theory Study
Source: Sci Rep. 2018 Apr 30;8:6702. doi: 10.1038/s41598-018-25027-1 (PMC5928166; doi:10.1038/s41598-018-25027-1)
Supplement: Supplementary file 1 — Supplementary information [file 41598_2018_25027_MOESM1_ESM.pdf]

Probing the Structural and Electronic Properties of Dirhenium Halide Clusters: A Density Functional Theory Study

Li Huan Zhang[1,2], Xin Xin Xia[1], Wei Guo Sun[1], Cheng Lu[2,3,*], Xiao Yu Kuang[1,*], Bo Le Chen[1] and George Maroulis[4,*]

[1]Institute of Atomic and Molecular Physics, Sichuan University, Chengdu 610065, China

[2]Department of Physics, Nanyang Normal University, Nanyang 473061, China

[3]Department of Physics and High Pressure Science and Engineering Center, University of Nevada, Las Vegas, Nevada 89154, United States

[4]Department of Chemistry, University of Patras, GR-26500 Patras, Greece

*Correspondence author. E-mail: lucheng@calypso.cn (Cheng Lu), scu_kuang@163.com (Xiao Yu Kuang), and maroulis@upatras.gr (George Maroulis)

clusters	Vibrational frequency
$Re_2F_8^{2-}$	55, 112, 112, 133, 150, 155, 155, 174, 198, 209, 218, 218, 218, 218, 234, 355, 470, 504, 508, 511, 511, 532, 532, 585
$Re_2Cl_8^{2-}$	44, 69, 107, 107, 107, 107, 112, 134, 136, 148, 148, 160, 160, 169, 176, 259, 262, 295, 306, 314, 314, 319, 319, 341
$Re_2Br_8^{2-}$	23, 37, 68, 68, 75, 76, 76, 91, 100, 100, 103, 103, 116, 116, 117, 147, 148, 187, 193, 214, 214, 216, 216, 311
$Re_2I_8^{2-}$	10, 21, 48, 48, 63, 66, 66, 76, 79, 79, 87, 92, 92, 92, 93, 94, 94, 134, 143, 164, 174, 174, 294

Table S1. The vibrational frequencies of lowest-energy $Re_2X_8^{2-}$ (X = F, Cl, Br, I) clusters.

$Re_2F_8^{2-}$				$Re_2Cl_8^{2-}$			
Re	0.00000000	0.00000000	1.09773886	Re	0.00000000	0.00000000	1.10479084
Re	−0.00000000	0.00000000	−1.09773886	Re	−0.00000000	0.00000000	−1.10479084
F	0.00000000	−1.90455601	−1.60263728	Cl	0.00000000	2.34659268	−1.72775235
F	−1.90455601	0.00000000	1.60263728	Cl	0.00000000	−2.34659268	-1.72775235
F	−1.90455601	−0.00000000	−1.60263728	Cl	2.34659268	0.00000000	−1.72775235
F	−0.00000000	1.90455601	−1.60263728	Cl	−2.34659268	−0.00000000	−1.72775235
F	1.90455601	0.00000000	−1.60263728	Cl	0.00000000	2.34659268	1.72775235
F	0.00000000	1.90455601	1.60263728	Cl	−0.00000000	−2.34659268	1.72775235
F	1.90455601	−0.00000000	1.60263728	Cl	2.34659268	−0.00000000	1.72775235
F	0.00000000	−1.90455601	1.60263728	Cl	−2.34659268	0.00000000	1.72775235
$Re_2Br_8^{2-}$				$Re_2I_8^{2-}$			
Re	−0.00000000	−0.00000000	−1.11076306	Re	−0.00000000	−0.00000000	1.11864110
Re	0.00000000	−0.00000000	1.11076306	Re	−0.00000000	−0.00000000	−1.11864110
Br	0.00000000	2.49684155	−1.81183386	I	0.00000000	2.67410643	1.93085633
Br	0.00000000	2.49684155	1.81183386	I	0.00000000	−2.67410643	1.93085633
Br	0.00000000	−2.49684155	−1.81183386	I	−2.67410643	−0.00000000	1.93085633
Br	−0.00000000	−2.49684155	1.81183386	I	2.67410643	0.00000000	1.93085633
Br	2.49684155	0.00000000	−1.81183386	I	−0.00000000	−2.67410643	−1.93085633
Br	2.49684155	−0.00000000	1.81183386	I	−0.00000000	2.67410643	−1.93085633
Br	−2.49684155	−0.00000000	−1.81183386	I	−2.67410643	0.00000000	−1.93085633
Br	−2.49684155	0.00000000	1.81183386	I	2.67410643	−0.00000000	−1.93085633

Table S2. Cartesian coordinates of optimized lowest-energy geometries of $Re_2X_8^{2-}$ (X = F, Cl, Br, I) clusters at the B3LYP level.

clusters		State	Sym.	ΔE	E_{gap}
$Re_2F_8^{2-}$	1a	$^1A_{1g}$	D_{4h}	0.00	1.94
	2a	^5A	C_2	1.14	1.50
	3a	$^5A'$	C_s	1.71	2.19
	4a	$^1A_1$	C_{2v}	3.36	1.90
$Re_2Cl_8^{2-}$	1b	$^1A_{1g}$	D_{4h}	0.00	1.71
	2b	^5A	$\mathrm{C_1}$	1.16	1.73
	3b	$^5A'$	C_s	1.22	1.59
	4b	$^5B_2$	C_{2v}	1.47	1.10
$Re_2Br_8^{2-}$	1c	$^1A_{1g}$	D_{4h}	0.00	1.66
	2c	$^3B_2$	C_{2v}	0.28	2.01
	3c	$^5B_1$	C_{2v}	1.31	1.51
	4c	$^1A_1$	C_{2v}	3.07	1.76
$Re_2I_8^{2-}$	1d	$^1A_{1g}$	D_{4h}	0.00	1.43
	2d	$^1A_1$	C_{2v}	0.43	1.02
	3d	$^5B_2$	D_{2d}	1.50	1.10
	4d	$^1A_1$	C_{2v}	2.43	1.61

Table S3. Electronic states, point group symmetries, relative energies ΔE (eV), HOMO−LUMO gaps E_{gap} (eV) of all low-lying $Re_2X_8^{2-}$ (X = F, Cl, Br, I) isomers.

cluster	B3LYP		BP86		Ref 2
	Re−Re	Re−X	Re−Re	Re−X	Re−Re
$Re_2F_8^{2-}$	2.20	1.97	2.22	1.98	2.19
$Re_2Cl_8^{2-}$	2.21	2.43	2.23	2.42	2.21
$Re_2Br_8^{2-}$	2.22	2.59	2.24	2.58	2.23
$Re_2I_8^{2-}$	2.24	2.79	2.26	2.78	2.25

Table S4. Re−Re and Re−X bond lengths (Å) of all lowest-energy $Re_2X_8^{2-}$ (X = F, Cl, Br, I) clusters calculated by different method.

clusters	ADE		VDE	
	Cal.	Exp.	Cal.	Exp.
$Re_2F_8^{2-}$	0.05		0.15	
$Re_2Cl_8^{2-}$	0.98	1.00	1.19	1.16
$Re_2Br_8^{2-}$	1.05		2.09	
$Re_2I_8^{2-}$	0.82		1.21	

Table S5. The calculated VDE and ADE values of the ground-state $Re_2X_8^{2-}$ (X = F, Cl, Br, I) clusters together with the available experimental data.

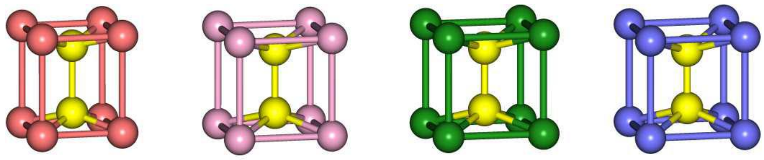

D_{4h} $Re_2F_8^{2-}$ | D_{4h} $Re_2Cl_8^{2-}$ | D_{4h} $Re_2Br_8^{2-}$ | D_{4h} $Re_2I_8^{2-}$

Figure S1. The geometrical structures of the ground states $Re_2X_8^{2-}$ (X = F, Cl, Br, I) clusters calculated by BP86 method.
